# Semaphorin Regulation by the Chromatin Remodeler CHD7: An Emerging Genetic Interaction Shaping Neural Cells and Neural Crest in Development and Cancer

**DOI:** 10.3389/fcell.2021.638674

**Published:** 2021-04-01

**Authors:** Antonella Lettieri, Roberto Oleari, Alyssa J. J. Paganoni, Cristina Gervasini, Valentina Massa, Alessandro Fantin, Anna Cariboni

**Affiliations:** ^1^CRC Aldo Ravelli for Neurotechnology and Experimental Brain Therapeutics, Università degli Studi di Milano, Milan, Italy; ^2^Department of Health Sciences, Università degli Studi di Milano, Milan, Italy; ^3^Department of Pharmacological and Biomolecular Sciences, Università degli Studi di Milano, Milan, Italy; ^4^Department of Biosciences, Università degli Studi di Milano, Milan, Italy

**Keywords:** CHD7, chromatin remodeler, semaphorins, development, cancer

## Abstract

CHD7 is a chromatin remodeler protein that controls gene expression *via* the formation of multi-protein complexes with specific transcription factors. During development, CHD7 controls several differentiation programs, mainly by acting on neural progenitors and neural crest (NC) cells. Thus, its roles range from the central nervous system to the peripheral nervous system and the organs colonized by NC cells, including the heart. Accordingly, mutated *CHD7* is linked to CHARGE syndrome, which is characterized by several neuronal dysfunctions and by malformations of NC-derived/populated organs. Altered CHD7 has also been associated with different neoplastic transformations. Interestingly, recent evidence revealed that semaphorins, a class of molecules involved in developmental and pathological processes similar to those controlled by CHD7, are regulated by CHD7 in a context-specific manner. In this article, we will review the recent insights that support the existence of genetic interactions between these pathways, both during developmental processes and cancer progression.

## Introduction

Gene expression regulation is essential for normal tissue and organ development and maintenance. Chromatin remodeling *via* the action of several enzymes plays key roles in the control of gene expression, mainly by affecting cellular proliferation and differentiation (Layman et al., 2010; Chen and Dent, 2014). One of the main aims of current developmental biology is to identify the molecular pathways that regulate chromatin structure and gene expression as well as to understand how such regulation influences organogenesis. Specifically, chromatin remodeling is crucial for most biological processes involving DNA, such as transcription, chromosome segregation, DNA replication, and repair (Clapier and Cairns, 2009). Not surprisingly, most chromatin remodelers are indispensable for normal development (Ho and Crabtree, 2010).

The chromodomain helicase DNA binding gene 7 (*CHD7*) regulates the transcription of tissue-specific target genes either alone or by stabilizing other transcription factor complexes (Schnetz et al., 2009, 2010; Bajpai et al., 2010; Zentner et al., 2010). During development, CHD7 downregulation leads to alterations in the expression of its target genes, thus affecting the formation of multiple organ systems. With similar mechanisms, in adults, CHD7 controls the expression of gene sets involved in stemness maintenance during homeostasis (Martin, 2010; Micucci et al., 2015) as well as in invasiveness and angiogenesis during cancer progression (Mills, 2017).

Among CHD7 target genes, a central role is emerging for members of the semaphorin family (Schulz et al., 2014b; Ufartes et al., 2018; Liu et al., 2019). Indeed several semaphorins have been shown to regulate aspects of development and pathology (Masuda and Taniguchi, 2015; Mastrantonio et al., 2021) that share several common traits with CHD7 biology. Thus, although the phenotypic effects of semaphorin signaling in this context are still largely unknown, investigating the relationship between CHD7 and semaphorins both during development and disease will be significant to provide novel insights into developmental and pathological dynamics. This will pave the way to the proposal of novel therapeutic strategies aimed at bypassing CHD7 dysregulation, both in developmental syndromes and cancer.

In this article, we will review the latest insights on CHD7 and semaphorins interactions during embryonic development, developmental disorders, and cancer progression, with a particular focus on neural crest (NC) and neuronal systems.

## Chromodomain Helicase DNA Binding Gene

### CHD7 Structure, Expression, and Function During Development

The *CHD7* gene is located on chromosome 8q12.2 (GRCh38 chr8:60,678,739–60,868,027) and contains 38 exons encoding for a large protein of 2,997 amino acids (approximately 336 kDa). CHD7 is a member of the CHD family of ATP-dependent chromatin remodelers, which hydrolyze ATP to regulate nucleosome assembly/organization (Clapier et al., 2017) and ultimately control gene expression. Similar to other CHD family members, CHD7 is composed of two helicase domains (helicase N, including DEXDc domain, and HELICc, containing an ATP binding site and a DNA binding domain), a SANT domain (SWI3, ADA2, N-CoR, and TFIIIB), and two BRK (Braham and Kismet) domains and uniquely possesses two chromodomain at its N-terminus (Clapier et al., 2017).

As a crucial epigenetic factor, CHD7 amino acid sequence is highly conserved across species from invertebrates to mammals (Hall and Georgel, 2007; Marfella and Imbalzano, 2007; Li and Mills, 2014). Most of the studies have focused on *Drosophila melanogaster* homolog gene *Kismet* and in *Xenopus laevis* (xenopus), *Danio rerio* (zebrafish), and *Mus musculus* (mouse). In vertebrates, the expression pattern of CHD7 is also conserved, with a broad distribution in the central nervous system (CNS) (Sanlaville et al., 2006) and in NC derivatives, including craniofacial cartilages, otic vesicle, heart, mesenteric nervous system, and cranial and olfactory nerves (Bosman et al., 2005; Patten et al., 2012; Pauli et al., 2017). Accordingly, CHD7 has essential functions in regulating some of the genetic programs and molecular mechanisms that control neuroectoderm- and NC-derived tissues, both during development and in adulthood (Layman et al., 2010). For example, CHD7 is essential for neural development and adult neural stem cell maintenance (Jones et al., 2015; Feng et al., 2017a,b; Chai et al., 2018) as well as for NC cell specification, migration, and differentiation (Bajpai et al., 2010; Fujita et al., 2014; Sperry et al., 2014).

**FIGURE 1:**
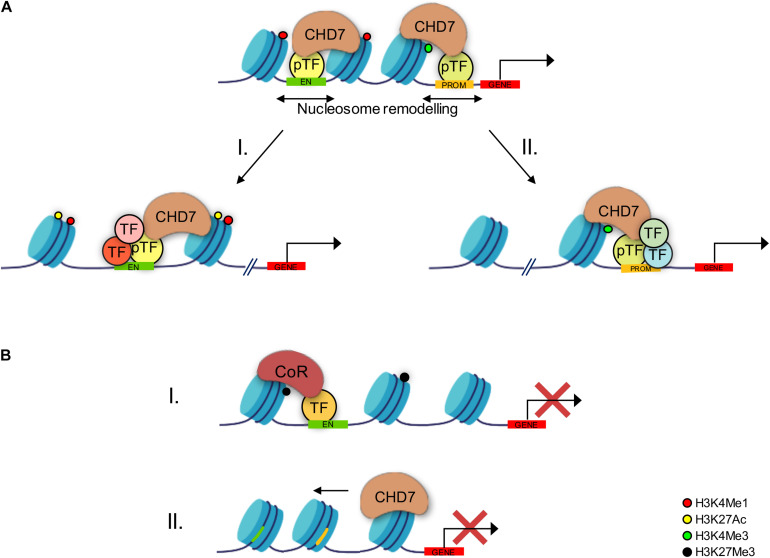
Schematic drawing showing the mechanisms through which CHD7 controls gene expression. **(A)** CHD7 is predominantly recruited at active enhancers (H3K4me1 mark, red dots) and, even if with less extent, promoters (H3K4Me3 mark, green dots) by the presence of pioneer transcription factors and specific histone methylations. The CHD7-induced chromatin opening *via* nucleosome remodeling allows regulatory elements to become more accessible to additional transcription factors (TFs) at both enhancers (I) and promoters (II). Furthermore, co-activators, such as histone acetylases, may be recruited as well at enhancer sites (I), thus promoting further histone modifications (H3K27ac, yellow dots) associated with enhanced transcription activity. **(B)** CHD7 can also act as transcription repressor. Chromatin remodeling might result in TFs complexed with co-repressors that promote repressive histone modification (H3K27me3, black dot) (I). Conversely, CHD7 might promote nucleosome repositioning, resulting in less accessible chromatin and in decreased transcriptional activity (II).

Mechanistically, genomic experiments based on chromatin immunoprecipitation (ChIP)-on-chip performed in different cellular models have shown that CHD7 localizes to discrete and cell type-specific locations along the chromatin. In particular, the cell-specific binding of CHD7 correlates with the pattern of histone H3 methylation at lysine 4 (H3K4me) or acetylation at lysine 27 (H3K27ac) (Schnetz et al., 2009, 2010; Micucci et al., 2015). Most of these CHD7 binding sites on chromatin locate distal to transcription start codons, a feature typical of enhancer elements (H3K4me1). At these sites, CHD7 binds in combination with other DNA-binding proteins, including p300, OCT4, SOX2, NANOG, SMAD1, and STAT3 (Martin, 2010). CHD7 binding sites have also been found, although at a lesser extent, in active promoter regions characterized by H3K4me3 signature (Schnetz et al., 2009, 2010). Furthermore, genome-wide expression studies in model systems have suggested that CHD7 can function as an activator or repressor of gene transcription (Schulz et al., 2014b). One possibility is that the activator or repressor activity of CHD7 at a particular regulatory element is dependent on local DNA context. Alternatively, CHD7 may function exclusively as an enhancer of gene expression, and increased gene expression in CHD7-deficient cells may be secondary to downregulated expression of transcriptional repressors (Basson and van Ravenswaaij-Arts, 2015; Pauli et al., 2017; Figure 1). For example, CHD7 cooperates with SMAD1 to positively regulate *Bmp4* expression during heart morphogenesis (Liu et al., 2014). CHD7 also positively regulates *Fgf10*, *Otx2*, and *Ngn1* expression during ear development (Hurd et al., 2010), whereas in the cerebellum, CHD7 depletion causes OTX2 upregulation and spatial expansion (Yu et al., 2013). Finally, different SOX family members are regulated by CHD7, including SOX2 in neural stem cells (NSCs) (Engelen et al., 2011) as well as SOX9 and SOX10 in NC cells (Bajpai et al., 2010; Asad et al., 2016; Fujita et al., 2016).

### CHD7 and CHARGE Syndrome

Several animal models have been established to study the genetic and molecular mechanisms through which CHD7 regulates development and homeostasis in various tissues. Since *Chd7*-null mice have embryonic lethality at around embryonic day (E) 10.5 (Hurd et al., 2007), heterozygous *Chd7* mouse mutants (Feng et al., 2017b) as well as genetically modulated xenopus and zebrafish embryos (Bajpai et al., 2010; Schulz et al., 2014b; Asad et al., 2016; Ufartes et al., 2018; Liu et al., 2019) have been widely used to study CHD7 biological function as well as to recapitulate different aspects of the clinical phenotypic spectrum of patients carrying CHD7 mutations. Specifically, heterozygous loss-of-function mutations in *CHD7* (MIM 608892) are the main cause of CHARGE syndrome (MIM 214800), a multisystemic developmental disorder characterized by the association of *c*oloboma, *h*eart defects, *a*tresia of choanae, *r*etarded growth, *g*enital defects, and *e*ar abnormalities (Vissers et al., 2004). A high variability in the phenotypic spectrum of the clinical traits is reported in CHARGE patients (Bergman et al., 2011; van Ravenswaaij-Arts and Martin, 2017). In this respect, it has been recently proposed that genes regulated by and/or in an epistatic relationship with CHD7 may represent “modifier” genes (Basson and van Ravenswaaij-Arts, 2015). Mutations or polymorphisms in these genes could conceivably be responsible for much of the clinical variation, for instance, by altering the penetrance and/or severity of specific disease traits in the context of *CHD7* haploinsufficiency (Basson and van Ravenswaaij-Arts, 2015). In addition, since CHD7 is thought to recruit cell type-specific enhancers to modulate transcription (Schnetz et al., 2009, 2010; Bajpai et al., 2010; Zentner et al., 2010), it is also possible that the clinical variability of the congenital anomalies in CHARGE syndrome might be due to alterations in transcription of tissue-specific genes normally regulated by CHD7 during development (Basson and van Ravenswaaij-Arts, 2015).

Furthermore, CHARGE syndrome shares some phenotypic overlap with other congenital disorders such as Kabuki (Schulz et al., 2014a) and DiGeorge syndromes (Randall et al., 2009) (for a summary of the overlapping syndromes discussed in this review, refer to Table 1). For example, it has been proposed that CHD7 and KMT2D (mutated in Kabuki syndrome) function in the same chromatin modification machinery, thus pointing out a mechanistic connection and offering a possible explanation for the phenotypic overlap between these two syndromes (Schulz et al., 2014a).

**TABLE 1:**
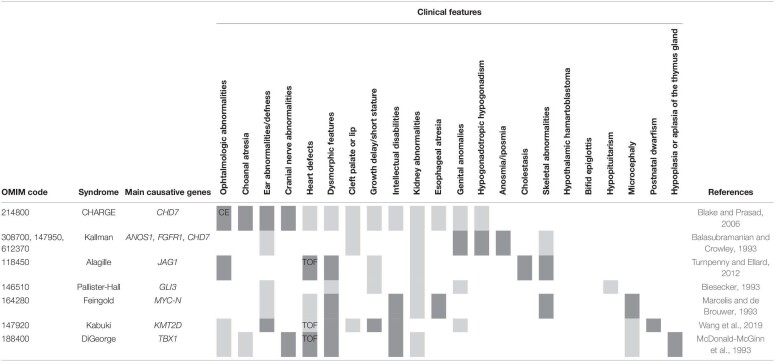
Different syndromes as discussed in the text, the main mutated genes, and the characteristic clinical features.

*Dark gray denotes the presence of the indicated symptom in each syndrome, and white denotes its absence (major features), whereas light gray indicates that the symptom is not always present (minor features). TOF, tetralogy of Fallot; CE, coloboma of the eye.*

### CHD7 and Cancer

In many forms of cancers, deregulation of CHD7 expression promotes oncogenesis. On one hand, mutations that activate CHD7 have been found in small cell lung cancer in response to tobacco smoke (Pleasance et al., 2010), and high CHD7 expression is also found in cutaneous T-cell lymphoma (Litvinov et al., 2014). On the other hand, low CHD7 levels correlate with an enhanced survival rate of patients with pancreatic tumor (Colbert et al., 2014), and loss-of-function mutations of *CHD7* are reported in colorectal as well as gastric cancer (Kim et al., 2011; Tahara et al., 2014). Furthermore, a recent large-scale genomic analysis of human cancers confirmed the dysregulation of CHD7 in many malignancies, including breast, lung, colorectal, and ovarian cancers (Chu et al., 2017). In particular, CHD7 overexpression in breast cancer is more prevalent in aggressive subtypes, where it is combined with the activation of *NRAS* and *MYCN* oncogenes and correlates with high tumor grade and poor prognosis; conversely, CHD7 depletion in human breast cancer cell lines is sufficient to inhibit cell proliferation and downregulate the expression of CHD7 target genes, including *NRAS* oncogene (Chu et al., 2017). In endometrial cancer, CHD7 modulates cancer-related pathways which have a negative impact on patient survival (Lu et al., 2020). Consistent with the role exerted during neural development, CHD7 is also implicated in tumors affecting CNS and NC-derived tissues, including medulloblastoma, glioblastoma, and neuroblastoma (Robinson et al., 2012; Badodi et al., 2017; Mondal et al., 2018; Boyd et al., 2019; Machado et al., 2019) (see Chapter 5).

## Semaphorins

### Semaphorin Structure, Expression, and Functions During Development

Semaphorins constitute a large family of glycoproteins bearing a distinctive SEMA domain which allows dimerization and receptor binding (Gherardi et al., 2004). Semaphorins are highly conserved among species and comprise eight classes: class V is peculiar to viruses, classes 1 and 2 are restricted to invertebrates, while classes 3–7 are found in vertebrates (Yazdani and Terman, 2006). Vertebrate semaphorins can be secreted (class 3A–G), transmembrane (classes 4A–G, 5A and B, and 6A–D), or membrane-anchored (class 7A) (Messina and Giacobini, 2013).

Semaphorins regulate cytoskeletal dynamics, thereby influencing cell shape, differentiation, motility, and survival. They exert their biological function by initiating an intracellular signaling cascade triggered by their binding to neuropilins (NRPs) and/or plexins (PLXNs) on the cell surface (Alto and Terman, 2017). Additionally, semaphorins can bind to other transmembrane proteins such as cell adhesion molecules, integrins, cadherins, or tyrosine-kinase receptors (e.g., VEGFR and Met) [detailed semaphorin structures and receptor interactions are reviewed in Alto and Terman (2017) and Messina and Giacobini (2013)].

Semaphorins are widely expressed in all tissues where they regulate many different developmental processes. In the nervous system, where they were first discovered, semaphorins are expressed by neurons and glia cells and act mainly as repulsive cues to regulate nervous system patterning through axon guidance and cell migration (Pasterkamp and Giger, 2009). For example, they are expressed in the striatum to prevent interneuron invasion (Marín et al., 2001) and in the spinal cord to control commissural axon midline crossing (Kolodkin and Tessier-Lavigne, 2011). In addition, molecular gradients of semaphorins restrict the migration and segregation of NC cells, which contribute to the formation of several organs including the peripheral nervous system (Theveneau and Mayor, 2012).

Besides repulsive activity, semaphorins play essential roles in cell attraction, proliferation, differentiation, and survival (Jongbloets and Pasterkamp, 2014); such different activities are thought to depend on the plethora of receptor complexes that they interact with and on the cellular contexts. For example, SEMA3E receptor PLXND1 is expressed by both hypothalamic GnRH neurons and axons of ventrolateral cortical and striatal neurons. Depending on cellular context, the SEMA3E–PLXND1 interaction is able to promote GnRH neuron survival (Cariboni et al., 2015) or cortical and striatal axon repulsion (Chauvet et al., 2007). The addition of NRP1 and VEGFR2 to PLXND1 in the receptor complex on subicular axons instead leads to a switch in the response to SEMA3E from repulsive to attractive (Chauvet et al., 2007). Semaphorins are also able to promote cell proliferation as shown by Berndt and Halloran (2006) in hindbrain neuroepithelial cells after SEMA3D knockdown.

It is now well established that semaphorins also regulate key developmental processes in the cardiovascular, immune, endocrine, hepatic, renal, reproductive, respiratory, and musculoskeletal systems (Jongbloets and Pasterkamp, 2014; Fard and Tamagnone, 2020) as well as being deregulated in cancer cells (Tamagnone, 2012; Rehman and Tamagnone, 2013; Fard and Tamagnone, 2020). Among non-nervous systems, semaphorins play pivotal roles in cardiovascular system development (Epstein et al., 2015), whose detailed description will be covered in section “CHD7 and Semaphorin Interactions in the NC.”

### Semaphorins and Disease

Semaphorins have also been implicated in pathological conditions such as cancer (Tamagnone, 2012; Ahammad, 2020) and developmental disorders (Fard and Tamagnone, 2020).

In cancer, semaphorins can play a role in tumor progression by sustaining cell proliferation, evasion of apoptosis, oxidative stress regulation, neo-angiogenesis, invasion and metastasis, pro-tumorigenic inflammation, and escaping the immune system surveillance (Rehman and Tamagnone, 2013; Ahammad, 2020). A semaphorins subset instead exhibits tumor-suppressive activity through different mechanisms such as reverse signaling, inhibition of matrix metalloproteinases or reduction of integrins, which both promote metastasis, or by modulating vascular endothelial growth factor (VEGF) signaling, which is a potent pro-angiogenic factor (Ahammad, 2020).

Recent evidence also suggests that mutations in genes coding for semaphorin signaling members may also be implicated in developmental genetic disorders. Mutations in *SEMA3A* and *SEMA3E* (Hanchate et al., 2012; Young et al., 2012; Känsäkoski et al., 2014; Cariboni et al., 2015) have been found in patients with Kallmann syndrome (KS), which is due to a defect in the migration of GnRH neurons during development and share some phenotypic features with CHARGE syndrome (Dodé and Hardelin, 2009) (see also section “GnRH Neurons”).

## CHD7 and Semaphorin Interactions

Since CHD7 and semaphorins share overlapping functions in the development of NC and neuronal cells, it is not surprising that the main associations/interactions between these two classes of molecules have been reported in tissues of neural and NC origin, both in physiological and pathological settings. Thus, in this second part of the review, we will focus our attention on the known CHD7–semaphorins interactions during CNS and NC development as well as in tumors derived from these tissues.

### CHD7 and Semaphorin Interactions in the CNS

Precise temporal and spatial control of gene expression is essential for brain development, and several evidence support key roles for CHD7 in orchestrating developmental dynamics underpinning brain development in different regions and at different stages (Goodman and Bonni, 2019). Accordingly, multiple structural defects in the brain of CHARGE patients have been reported, such as hypoplasia of olfactory bulb and cerebellum, agenesis of the corpus callosum, and microcephaly and atrophy of the cerebral cortex (Feng et al., 2017b). Consistent with defects in brain anatomy, intellectual deficiency, although of different severity, has been consistently reported in several CHARGE patients (Bergman et al., 2011).

In mouse, CNS development starts as early as E8.5 when the neural tube starts folding and continues postnatally until postnatal day (P) 21 (Chen et al., 2017). Within this time window, neural precursors first proliferate, then migrate, and differentiate into different neuronal cell types. Neuron production commences at approximately the time when neural tube closure is almost complete, as early as E9, and progresses through early postnatal life. Although CNS development as an organ system spreads over a long period, each population of neurons has generally a fairly tight developmental window when they may be produced (Chen et al., 2017).

CHD7 expression can be found during the early patterning of the brain (Hurd et al., 2007; Jiang et al., 2012), which occurs in mouse between E10 and E12.5 (Chen et al., 2017). Accordingly, heterozygote *Chd7*^*Gt/+*^ mouse embryos exhibited delayed turning, while lethal homozygotes showed hypoplasia of the neuroepithelium, olfactory pit, and hindlimb and thickened but small in size otocyst (Hurd et al., 2007). At later stages, between E12.5 and E14.5 in mice, CHD7 is highly expressed in proliferating areas of the brain, such as the frontal cortex, the medial ganglionic eminence, the ventricular zone of medulla, and the external granule zone of cerebellum (Bosman et al., 2005; Sanlaville et al., 2006; Hurd et al., 2007), where it promotes neuronal differentiation of several cell types, including murine embryonic stem cells (Yao et al., 2020) and neural stem cells (Engelen et al., 2011), inner ear neuroblasts (Hurd et al., 2010), and human neuroepithelial cells (Chai et al., 2018). Conversely, low CHD7 expression was reported in differentiated areas of the brain (Hurd et al., 2007). During late embryogenesis and postnatal development, CHD7 promotes proliferation, self-renewal, and neurogenesis by inducing the transition from neural progenitors (i.e., cells of the CNS that give rise to many, if not all, of the glial and neuronal cell types that populate the CNS; Martínez-Cerdeño and Noctor, 2018) to neuroblasts (i.e., post-mitotic cells that do not divide further, Zhang and Jiao, 2015) in the inner ear and in the subventricular zones of lateral ventricles (Micucci et al., 2014).

CHD7 expression persists also in the adult brain within the neurogenic niches of the subgranular zone in the hippocampal dentate gyrus (Jones et al., 2015) and in the cerebellum (Feng et al., 2017a), where it contributes to the differentiation of adult hippocampal stem cells (Feng et al., 2013) and granule cells of the cerebellum (Whittaker et al., 2017), respectively.

In the next sections, we will discuss the interactions between CHD7 and semaphorins in the CNS.

#### Neural Stem Cells

**FIGURE 2:**
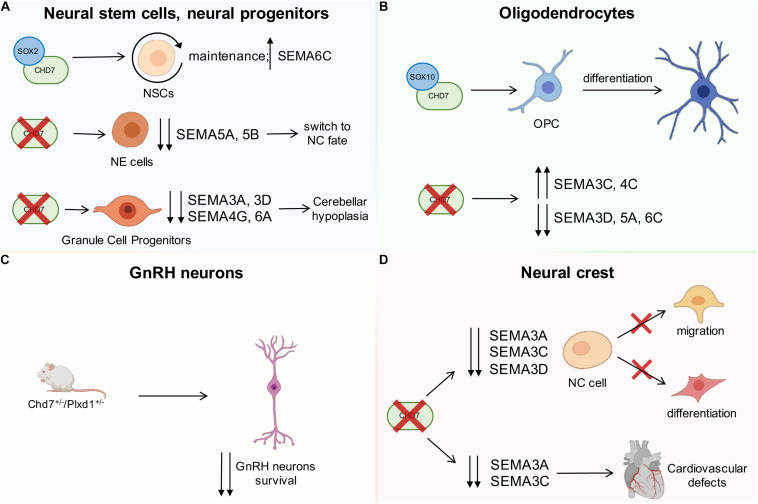
Schematic drawing showing the interactions between CHD7 and semaphorins during development. **(A)** CHD7 interacts with SOX2 transcriptional cofactor, which is essential for neural stem cell maintenance, and the expression of SEMA6C is directly controlled by CHD7/SOX2 (upper part of the drawing). Insufficient CHD7 activity in neuroepithelial cells leads to downregulation of the indicated semaphorins and switch to a neural crest (NC) fate (middle drawing). In the cerebellum (bottom), CHD7 depletion in granule cell progenitors reduces the expression of the indicated semaphorins and leads to hypoplasia. **(B)** CHD7 is expressed in the oligodendrocyte lineage of the brain, and CHD7/SOX10 interaction leads to the differentiation of oligodendrocyte precursor cells (OPCs). CHD7 depletion in OPCs induces the upregulation of SEMA3C and 4C and the downregulation of SEMA3D, 5A, and 6C. **(C)** Compound heterozygous mice for the SEMA3E-receptor PLXND1 and CHD7 display a more significant loss of GnRH neurons compared to single heterozygous mutants. **(D)** CHD7 is highly expressed in NC pre-migratory cells, and its depletion causes the downregulation of SEMA3A, 3C, and 3D, which are known to regulate cell migration and differentiation. CHD7 is also expressed by a subset of NC cells that participate in the development of the heart. In this context, diminished SEMA3A and SEMA3C expression due to the absence of CHD7 leads to cardiovascular defects. Red crosses indicate a lack of the indicated gene or impairment of the indicated biological process; the up and down arrows indicate expression changes of the indicated genes.

CHD7 was identified as a transcriptional cofactor of SOX2 (Engelen et al., 2011), which is essential for the maintenance of neural stem cells (i.e., self-renewing multipotent cells able to give rise to neurons and glia cells of the nervous system; Martínez-Cerdeño and Noctor, 2018). In agreement, the expression pattern of genes in NSCs after shRNA-mediated knockdown of *Sox2* or *Chd7* is similarly misregulated. Hence, SOX2 and CHD7 are correlated and co-regulate a set of common target genes including *JAG1*, *GLI3*, and *MYCN*, which are mutated in different forms of cancers as well as in Alagille, Pallister-Hall, and Feingold syndromes, that share some clinical traits with CHARGE (Basson and van Ravenswaaij-Arts, 2015; Pauli et al., 2017; see also Table 1). In addition, the expression of the semaphorin member *Sema6c*, which is broadly distributed in the mammalian brain (Qu et al., 2002), is directly controlled by CHD7/SOX2, which bind to the promoter region of this gene (Figure 2). However, CHD7 displays more genomic binding sites than SOX2, suggesting the possible involvement of additional transcription factors in CHD7-mediated gene regulation. Among them, CHD7 binds to several semaphorin loci in NSCs, including enhancer regions of *Sema3b*, *3f*, *3g*, *5a*, *5b*, and *6a* and promoters of *Sema3a*, *4c*, *5b*, *6a*, *6c*, and *6d*. Whether CHD7 alone is sufficient to regulate gene expression of these semaphorins or additional transcription factors are required is still unknown (Engelen et al., 2011).

More recently, CHD7 was also demonstrated to be highly expressed and to be required for the maintenance of cell identity in human induced pluripotent stem cell-derived neuroepithelial (iPSC-NE) cells, which are early NSCs that can further differentiate into radial glial cells (i.e., bipolar-shaped neural progenitor cells that can, in turn, produce both neurons and glial cells, including astrocytes and oligodendrocytes; Falk et al., 2012). Genome distribution analysis revealed that CHD7 is predominantly located at active enhancer elements as it overlaps with p300, H3K27ac, and H3K4me1 occupancy, and CHD7-bound genes enriched neurulation pathway, consistent with CHARGE syndrome features. Among these active enhancers, distal regions of *SEMA3A*, *3C*, *5B*, and *6A* were occupied by CHD7; however, only *SEMA5B* was downregulated in CHD7-depleted and CHARGE patients-derived NE cells (Chai et al., 2018). Interestingly, the CHD7 target SEMA5B has been previously associated with synaptogenesis and axon elongation, and its mutation is implicated in several forms of neurodevelopmental disorders, including autism (O’Connor et al., 2009). As a result of the global transcriptomic changes induced by insufficient CHD7 activity, neural progenitors switch to a NC fate (Chai et al., 2018).

#### Cerebellar Neural Progenitors

CHD7 is also required during the development of the cerebellum, and its role differs with developmental stages. During early embryonic development, CHD7 is essential for the maintenance of rhombomere 1 identity and for OTX2/FGF8 expression in the isthmus organizer, with disruption of the latter predisposing the embryo to cerebellar vermis hypoplasia (Yu et al., 2013). Perinatally, CHD7 is predominantly expressed in granule cell progenitors, and its depletion in *Atoh1*-Cre;*Chd7*^*flox/flox*^ mice reduces the expression of several semaphorins, including SEMA3A, 3D, 4G, and 6A, and leads to hypoplasia due to decreased proliferation (Feng et al., 2017a; Whittaker et al., 2017). In a *Ptf1a*-Cre;*Chd7*^*flox/flox*^ mouse line used to selectively inactivate CHD7 in neuronal progenitors that will differentiate into GABAergic Purkinje cells (Hoshino et al., 2005), no obvious cerebellar developmental defects are found instead, excluding an essential role of CHD7 in Purkinje cell lineage (Feng et al., 2017a).

#### Oligodendrocytes

Within the neural lineage, CHD7 is broadly expressed in oligodendrocytes (OL) of the brain and their progenitors (He et al., 2016). Similar to cerebellar granule cells, the expression of CHD7 in differentiated oligodendrocytes is higher than in oligodendrocyte precursor cells (OPCs) (Feng et al., 2017b). The latter is the main proliferative cell type in the adult brain, and the imbalance between OPC proliferation and differentiation can lead to either glioma development or impaired (re)myelination (Marie et al., 2018). Specifically, CHD7-driven chromatin opening in OPCs results in the expression of pro-differentiation genes such as *Sox10*, *Nkx2.2*, and *Gpr17* genes. On the other hand, OPC survival is promoted by CHD7-driven chromatin closing and the repression of the pro-apoptotic gene *p53*/*Trp53* (Marie et al., 2018). Interestingly, ChIP-sequencing analyses had shown that CHD7 binds several SOX10/OLIG2-active enhancers (defined by H3K27ac mark) both in OPCs and OLs, while it preferentially binds promoter regions (H3K4me3/H3K27ac) in OPCs (Marie et al., 2018). The induced *Chd7* loss-of-function in OPCs revealed that most genes downstream of CHD7 resulted to be upregulated rather than downregulated, suggesting that CHD7 mostly represses gene transcription despite its commonly known role as transcription activator (Schnetz et al., 2010; Marie et al., 2018). Among the genes that CHD7 directly modulates by binding to regulatory elements, *Sema3d, 5a*, and *6c* are downregulated, while *Sema3c* and *4c* are upregulated upon *Chd7* loss (Marie et al., 2018; Figure 2). Interestingly, SEMA5A was previously found to be expressed by OPCs and OL and demonstrated to inhibit the regeneration of retinal ganglion cells’ axon growth after injury *in vitro* (Goldberg et al., 2004). In addition, in the context of axon injury, it has been shown that OLIG2 binds enhancer elements of many semaphorin genes, promoting their activation with consequent suppression of axon regrowth (Ueno et al., 2020). Lastly, CHD7/SOX10 interaction selectively activates myelination-related genes during OL differentiation, suggesting this as a possible mechanism underlying the defects in cerebral white matter volume observed in CHARGE patients (He et al., 2016). Unlike OLIG2, the effective cooperation of SOX10 in regulating semaphorin expression is not established. However, CHD7, together with BRG1 and OLIG2, has been demonstrated to coordinate SOX10 activity during OL differentiation (He et al., 2016; Gregath and Lu, 2018; Parras et al., 2020). Moreover, both SOX10 and semaphorins are associated to OL differentiation program and downregulated upon CHD7 loss (Marie et al., 2018). Overall, these observations suggest a modulation of semaphorin expression downstream of CHD7/SOX10.

#### GnRH Neurons

GnRH neurons are neuroendocrine cells located in the hypothalamus where they produce and release the sex hormone GnRH, which is essential to activate and maintain the reproductive axis in all mammals (Herbison, 2016). During development, GnRH neurons are born in the nasal placode by ectoderm- and NC-derived cell intermixing (Forni and Wray, 2015). From there, they migrate along the terminal nerve to enter the forebrain, then scatter in the hypothalamus to attain their final positions (Taroc et al., 2017; Oleari et al., 2019a), and finally send projections to the median eminence, where GnRH is secreted (Wierman et al., 2011).

Semaphorins and their receptors have been shown to play crucial roles during both GnRH neuron development and GnRH secretion by modulating multiple processes (Messina and Giacobini, 2013; Lettieri et al., 2016; Oleari et al., 2019b). For example, class 3 SEMA3A has been shown to control terminal nerve patterning (Cariboni et al., 2011; Oleari et al., 2019a) as well as to control median eminence plasticity (Giacobini et al., 2014), which are pivotal for the correct patterning of GnRH neurons; SEMA3E, *via* its PLXND1 receptor, directly promotes GnRH neuron survival in the brain (Cariboni et al., 2015; Figure 2); SEMA3G can interfere with other class 3 semaphorins, ultimately disrupting GnRH neuron migration (Oleari et al., 2020); SEMA4D and SEMA7A influence GnRH neuron migration in the nasal compartment (Giacobini et al., 2008; Parkash et al., 2012), with SEMA7A playing an additional role also in GnRH neuron axon elongation (Parkash et al., 2015).

Accordingly, several mutations in genes encoding for semaphorins and their receptors have been found in patients affected by hypogonadotropic hypogonadism and KS, which are genetic disorders characterized by GnRH deficiency with consequent absent or delayed puberty (Hanchate et al., 2012; Young et al., 2012; Känsäkoski et al., 2014; Cariboni et al., 2015; Marcos et al., 2017; Kotan et al., 2019, 2021).

Interestingly, mutations in *CHD7* have also been found in patients with KS (Kim et al., 2008; Cariboni et al., 2015), suggesting that both CHARGE syndrome and KS might share common genetic determinants and causes. Whereas nonsense mutations are frequent in CHARGE syndrome patients with severe phenotypes, missense mutations are associated with milder CHARGE syndrome and KS cases (Bergman et al., 2012). Notably, family members with identical *CHD7* mutations can present different levels of disease severity (Jongmans et al., 2009). This could be due to the presence of additional mutations in other genes with an epistatic relationship to *CHD7* (Basson and van Ravenswaaij-Arts, 2015). The genes encoding class 3 semaphorins and their receptors are good candidates for genetic CHD7 interactors in the GnRH system. First, *SEMA3E* mutations have been reported in a CHARGE syndrome patient (Lalani et al., 2004) presenting bilateral choanal atresia, cranial nerve dysfunction, genital hypoplasia, developmental delay, and growth retardation (Martin et al., 2001). *SEMA3E* mutations were also reported alongside *CHD7* mutations in two brothers with KS phenotype instead (i.e., hypogonadism and anosmia) (Cariboni et al., 2015). Accordingly, *Chd7–Plxnd1* compound heterozygous mice display a more severe neuronal GnRH phenotype than single heterozygous mutants (Figure 2; Cariboni et al., 2015), indicating that the two pathways are genetically interacting. Second, *CHD7* regulates *SEMA3A*, which encodes a guidance cue essential for the migration of NC cells (Schulz et al., 2014b; Ufartes et al., 2018; see below) and for the patterning of the terminal nerve, which is used as a migratory scaffold by GnRH neurons. Accordingly, *Sema3a*^–/–^ mice display KS symptoms (Cariboni et al., 2011), and *SEMA3A* mutations can occur in patients with KS (Hanchate et al., 2012; Young et al., 2012; Känsäkoski et al., 2014) or CHARGE syndrome (Schulz et al., 2014b; Ufartes et al., 2018).

### CHD7 and Semaphorin Interactions in the NC

NC cells are a population of pluripotent migratory cells that arise from the neural plate border during early embryogenesis (starting at E8.5 in mice) and give rise to neural, skeletal, dermal, and mesenchymal structures, including cardiac, mesenteric, and cranial nerves (Gammill and Bronner-Fraser, 2003). The induction of NC involves several signaling molecules including retinoic acid, BMP, WNT, and FGF, which in concert lead to the activation of a gene regulatory network that includes the specifier genes SOX9 and SOX10 (Asad et al., 2016; Fujita et al., 2016). These genes regulate processes such as delamination, migration, and differentiation of NC cells (Pauli et al., 2017). Okuno et al. (2017) recently confirmed that CHARGE syndrome pathogenesis includes defective NC cell migration. In particular, induced pluripotent stem cells (iPSCs) generated from CHARGE syndrome patient-derived fibroblasts and differentiated into NC cells displayed multiple functional abnormalities, such as defects in delamination, migration, and motility (Okuno et al., 2017). These observations therefore confirm previous suggestions that the CHARGE syndrome belongs to the group of neurocristopathy diseases.

CHD7 is highly expressed in NC pre-migratory cells (Fujita et al., 2014) and is crucial to stimulate NC induction and to maintain multipotency as revealed by gene knockdown experiments in human embryonic stem cells (Bajpai et al., 2010; Schnetz et al., 2010). Moreover, CHD7 depletion in xenopus, zebrafish, and mouse embryos also leads to abnormalities in NC cell migration (Randall et al., 2009; Bajpai et al., 2010; Sperry et al., 2014; Asad et al., 2016).

Semaphorins are also known to be fundamental for NC contribution to embryonic development. In xenopus, *sema3a* expression guides the paths of cranial NC cells by being expressed in a stripe along the migrating cranial NC cells expressing a twist (Koestner et al., 2008). In mouse, SEMA3A and SEMA3F act as repulsive cues *via* PLXNA4/NRP1 and PLXNA3/NRP2, respectively, to direct cranial NC cells and ultimately contribute to cranial gangliogenesis and the organization of sensory neuron axonal projections within the facial nerve (Schwarz et al., 2008). Similarly, SEMA3A and SEMA3F signaling through NRPs is also required for correct patterning of dorsal root ganglia and sympathetic nervous system, which are derived from trunk NC cells (Kawasaki et al., 2002; Schwarz et al., 2009b, a; Maden et al., 2012). Moreover, SEMA3D is essential for proper migration and proliferation of NC cells emerging from the hindbrain region, and its depletion leads to defects in a subset of NC cell derivatives, including craniofacial cartilages and pigment cells in zebrafish (Berndt and Halloran, 2006).

A genome-wide transcriptomic analysis on *Chd7*^*whi/whi*^ mutant mouse embryos lacking functional CHD7 confirmed the direct functional relationship between semaphorin genes and CHD7 in NC cells. In these mice, CHD7 depletion causes the downregulation of class 3 semaphorins *Sema3a*, *Sema3c*, and *Sema3d* genes (Schulz et al., 2014b), which are known to regulate NC cell migration and differentiation (Yu and Moens, 2005; Berndt and Halloran, 2006; Sato et al., 2006; Toyofuku et al., 2008; Maden et al., 2012; Figure 2). The relationship between CHD7 and SEMA3A in NC development was confirmed by experiments of *chd7* knockdown in xenopus embryos, in which morphants exhibited an abnormal *sema3a* expression pattern (Schulz et al., 2014b). A few years later, xenopus was also used to demonstrate that *sema3a* knockdown phenocopied, although less severely, CHD7 silencing, leading to severe malformations of the craniofacial cartilage, coloboma of the eyes as well as heart defects. In addition, *sema3a* overexpression rescued the abnormal phenotype of *chd7*-depleted embryos, confirming that SEMA3A acts downstream of CHD7 (Ufartes et al., 2018). Another member of the class 3 semaphorins, SEMA3E, has recently been implicated in the NC defects that are found in CHARGE syndrome. In the zebrafish *chd7* mutant that models CHARGE syndrome, sema3e knockdown resulted in severe craniofacial malformations, including small eyes, defective cartilage, and an abnormal number of otoliths. By taking advantage of time-lapse imaging, Liu et al. additionally showed that both *sema3e* and *chd7* knockdown severely impaired cranial NC cell migration in the *sox10:EGFP* NC cell reporter transgenic fish line. *Sema3e* overexpression also rescued *chd7* depletion, suggesting a possible regulation of SEMA3E by CHD7 (Liu et al., 2019).

#### Cardiac NC and Heart Development

A subset of NC cells delaminates from the hindbrain region of the neural tube, between the otic vesicle and the third somite, and participates to the development of the heart. In particular, in mammals, NC cells regulate the septation of the outflow tract (OFT) into the base of the pulmonary artery and aorta as well as the remodeling of the pharyngeal arch arteries that contribute to the formation of the aortic arch. For these reasons, such subset of NC cells is defined as cardiac NC. Accordingly, defective cardiac NC cell function is a common cause of life-threatening congenital heart defects (Creazzo et al., 1998; Keyte and Hutson, 2012), such as the 22q11.2 deletion syndrome (also known as DiGeorge syndrome) and also the CHARGE syndrome.

Migrating cardiac NC cells express the semaphorin receptors PLXNA2, PLXND1, and NRPs (Toyofuku et al., 2008; Plein et al., 2015), and semaphorin signaling has been shown to contribute to cardiac NC cell-mediated remodeling of the heart. The membrane-bound SEMA6A and SEMA6B are expressed in the dorsal neural tube and the lateral pharyngeal arch mesenchyme as contact repellents along the cardiac NC cell migratory routes (Toyofuku et al., 2008). Perturbed expression of their PLXNA2 receptor blocks correct NC cell migration into the cardiac outflow tract in the developing chick embryo, and accordingly, *Plxna2*-deficient mouse embryos exhibit defective cardiac outflow tract formation (Toyofuku et al., 2008). These defects include common arterial trunk (CAT; also known as persistent truncus arteriosus), caused by failed OFT septation, and interrupted aortic arch (IAA; also referred to as a type b interruption), due to the regression of the left fourth pharyngeal arch artery. In addition, SEMA3C is secreted within the OFT and pharyngeal arches at the time when cardiac NC cells migrate into these tissues, and loss of SEMA3C causes CAT and IAA (Feiner et al., 2001). It has initially been proposed that SEMA3C acts as an attractive signal for cardiac NC cells as knockdown of its receptor NRP1 in cardiac NC cells perturbs their migration in chick embryos (Toyofuku et al., 2008). However, we more recently showed in mouse that cardiac NC cells do not respond to SEMA3C but rather provide an essential source of SEMA3C themselves (Plein et al., 2015). Thus, NC cell-derived SEMA3C signals to the NRP1-expressing OFT endothelium, thereby promoting endothelial-to-mesenchymal transition (endoMT). Such endoMT is then essential to supply cells to the endocardial cushions and reposition cardiac NC cells within the OFT to ensure correct septation (Plein et al., 2015). Recent lineage tracing experiments in chick and zebrafish embryos have shown that cardiac NC cells can also give rise to mature cardiomyocytes that are required for successful repair and regeneration of injured hearts, even though in mice this contribution is still disputed (George et al., 2020). Notably, SEMA3D has been shown to be expressed by cardiac NC cells in zebrafish, and reduced SEMA3D expression levels lead to a decrease in the number of cardiomyocytes and consequent congenital heart defects, including hypertrophic cardiomyocytes, decreased ventricular size, and defects in trabeculation (Sato et al., 2006). Finally, cardiac NC cells further contribute significantly to the autonomic innervation (sympathetic and parasympathetic) of the heart as well as to the cardiac conduction system (George et al., 2020). In this context, SEMA3A acts as a chemorepellent *via* NRP1 to create an epicardial-to-endocardial sympathetic innervation patterning that is critical to maintain a normal, arrhythmia-free heart rate in the mouse (Ieda et al., 2007; Maden et al., 2012; Figure 2).

Morbidity and mortality in CHARGE syndrome patients carrying loss-of-function mutations in *CHD7* are frequently caused by heart defects, including OFT and atrioventricular septal defects (Corsten-Janssen and Scambler, 2017). Mouse studies have shown that CHD7 has a role in several cell lineages during heart development, including the pharyngeal surface ectodermal (PSE) and the first and second heart field (Corsten-Janssen and Scambler, 2017), while its function in cardiac NC cells has been more controversial. In fact, despite the cranial abnormalities observed in NC cell-specific knockouts of *Chd7*, no cardiac defects were reported (Sperry et al., 2014), and rescue of CHD7 expression in NC cells failed to correct the pharyngeal arch artery abnormalities induced by the presence of only one functional *Chd7* allele (Randall et al., 2009). However, a recent report using different *Cre* and floxed lines suggests that CHD7 is required cell-autonomously in cardiac NC cells during heart development (Yan et al., 2020). Mechanistically, CHD7 has been proposed to genetically interact with *Tbx1*, the gene most often lost in 22q11.2 chromosome deletions causing DiGeorge syndrome (Corsten-Janssen and Scambler, 2017). As TBX1 is required in the PSE (Calmont et al., 2009) and known to recruit histone methyltransferases to chromatin (Fulcoli et al., 2016), such chromatin modifications could be recognized by CHD7 to remodel the nucleosomes at these sites.

Payne et al. (2015) unveiled an additional role of CHD7 in cardiac progenitors from both the first and second heart field during cardiovascular development. In fact, conditional ablation of *Chd7* in *Mesp1*-expressing anterior mesoderm resulted in cardiovascular defects akin to those seen in CHARGE patients as well as in a striking loss of cardiac innervation and embryonic lethality (Payne et al., 2015). In agreement with diminished *Sema3a* and *Sema3c* expression in whole E9.5 *Chd7*^–/–^ embryos (Schulz et al., 2014b), such defects were associated with downregulation of *Sema3a* in myocardial trabeculae and of *Sema3c* in both the second heart field and invading NC cells. Here CHD7 is thought to directly regulate transcription by binding the *Sema3c* promoter and modifying the local chromatin structure (Payne et al., 2015). Moreover, CHD7 further promotes semaphorin signaling by associating with BRG1 on the *Plxna2* promoter to activate the expression of PLXNA2 to guide cardiac NC cells into the OFT (Li et al., 2013).

## CHD7 and Semaphorin Interactions in Neuron- and NC-Derived Tumors

**FIGURE 3:**
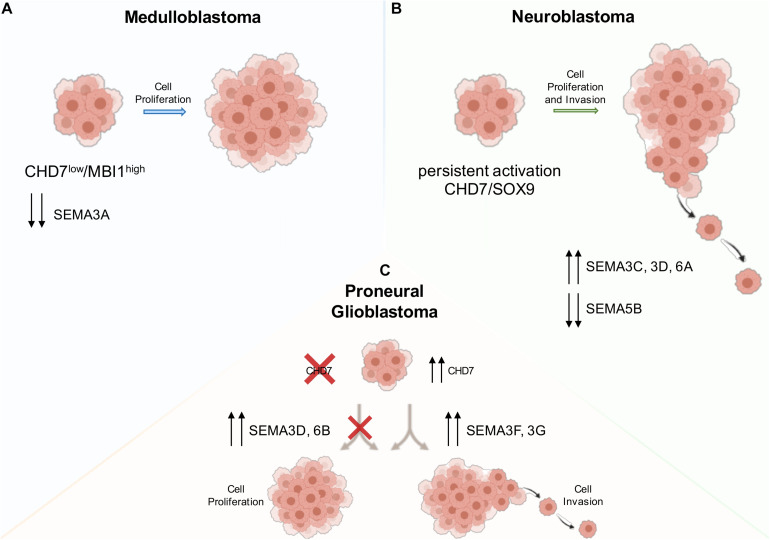
Schematic drawing showing the interaction between CHD7 and semaphorins in the indicated forms of tumors and its effects on tumor growth and invasiveness. **(A)** In medulloblastoma cells, a low expression of CHD7 and the subsequent downregulation of SEMA3A lead to increased cancer cell proliferation and higher tumor growth rate. **(B)** Neuroblastoma cells display increased proliferation and invasion due to a persistent activation of CHD7/SOX9 signaling, causing increased levels of NC-related genes, and decreased levels of neuronal differentiation genes. **(C)** Growth rate and invasion are affected in proneural glioblastoma cells by either CHD7 deletion or overexpression, which upregulate class 3 semaphorin downstream genes. Red crosses indicate a lack of the gene or impairment of the indicated biological process; the up and down arrows indicate expression changes of the indicated genes.

Loss- and gain-of-function mutations and copy number variations in *CHD7* have been found in several forms of cancer according to The Cancer Genome Atlas (TCGA) databases^1^; however, the mechanisms through which CHD7 acts during tumorigenesis, including the role of the controlled genes, have been poorly elucidated. Recently, a few efforts tried to dissect how CHD7 expression or deregulation in CNS- and NC-derived cancers affects tumorigenesis (Figure 3). These include reports on medulloblastoma, neuroblastoma, and glioblastoma. Consistent with the overlapping roles of semaphorins and CHD7 in NC and neuronal cell development, several links between CHD7 and semaphorins can be found in these tumors, which we will dissect in the next sections. Thus, deeper studies aimed at dissecting the mechanisms through which CHD7 controls semaphorin signaling in these diseases are also needed.

### Medulloblastoma

Medulloblastoma comprises a biologically heterogeneous group of embryonic/pediatric tumors of the cerebellum. Four main distinct subgroups of MB have been classically described (WNT, SHH, group 3, and group 4) depending on their molecular signature, each of which is associated with different onset and prognosis (Kool et al., 2012). Multiple recent reports have defined further intra-subgroup heterogeneity, hence increasing the number of biologically and clinically relevant subtypes to 12 subgroups (Cavalli et al., 2017; Northcott et al., 2017; Sharma et al., 2019). MB is the most common type of brain malignant tumor of childhood and the most common cause of pediatric death by cancer. Despite the fact that multimodal treatments have improved survival, relapse occurs in about one-third of patients (Northcott et al., 2019).

The least aggressive WNT and SHH MBs derive from constitutive activation of WNT and SHH signaling pathways in dorsal brainstem and granule cell precursors, respectively (Leto et al., 2016). Groups 3 and 4 MBs are still poorly characterized, but an involvement of cerebellar stem cells has been postulated. In particular, group 3 MB expresses high levels of *MYCN* (N-myc) and stemness markers, such as *SOX2*, *SOX9*, and *NES* (Nestin) (Kool et al., 2012). In contrast, little is known about group 4 MB even though they represent ∼30% of overall cases and are associated with the worst prognosis (Ramaswamy et al., 2016). *OTX2* overexpression, low *MYCN* levels, and presence of isochromosome 17 have been described in this subgroup of MB (Leto et al., 2016).

Recently, it has been shown that OTX2 promotes cancer stem cell self-renewal and increases tumor growth. Interestingly, OTX2 expression negatively correlates with several axon guidance genes, including semaphorins and their receptors, which result upregulated upon *OTX2* knockdown. In this context, ChIP-seq experiments have highlighted the presence of several OTX2 binding sites in genes that belong to the semaphorin pathway, such as *SEMA4D*, *SEMA6A*, *NRP1*, and *PLXNA2*, suggesting a direct regulation of semaphorin genes in MB (Stromecki et al., 2018). Consistent with these findings, group 4 MB patients with low SEMA4D expression are associated with poorest clinical outcomes, suggesting that OTX2 downstream genes, such as *SEMA4D*, could serve as putative prognostic genes for group 4 MB (Stromecki et al., 2018). Notably, OTX2 has been linked to CHD7 in pathogenetic mechanisms common to both KS and CHARGE syndromes (Pauli et al., 2017) as well as cerebellar development (Yu et al., 2013).

*CHD7* somatic mutations were found in groups 3 and 4 MB (Robinson et al., 2012), which showed lower CHD7 expression when compared to other MB subtypes. Genome-wide *in vivo* insertional mutagenesis screening revealed that CHD7 loss is frequently found in group 4 MB together with a high expression of the Polycomb gene BMI1. Furthermore, CHD7^*low*^/BMI1^*high*^ patients had a significantly worse overall survival compared to patients lacking this expression signature. This is due to increased cancer cell proliferation and higher tumor growth rate, consistent with CHD7/BMI1 cooperation in regulating ERK1/2-induced MB proliferation (Badodi et al., 2017). In the same study, the transcriptome of group 4 MB cells with a BMI1^*high*^; CHD7^*low*^ signature was compared to that of group 4 MB cells upon *BMI1* knockdown in a *CHD7*-silenced context. *SEMA3A* appeared downregulated in both conditions (Figure 3; Badodi et al., 2017). This finding raises the possibility that SEMA3A expression might be regulated by CHD7 at the onset of MB. Such possibility is consistent with previous findings about the deregulation of SEMA3A in cerebellar granule cell lacking CHD7 (Whittaker et al., 2017). However, further studies will be necessary to elucidate whether SEMA3A may determine a more aggressive phenotype in group 4 MB cells.

### Neuroblastoma

Neuroblastoma is the most common extracranial cancer in children and represents the cause of death in 10% of pediatric cancers. NBM patients are classified into low-, intermediate-, and high-risk groups according to disease stage, patient age, and specific genetic mutations. Developmentally, most NBMs originate from NC cells and are undifferentiated tumors, consisting of small round neuroblasts mainly localized in sympathetic ganglia or in the adrenal medulla (Brodeur, 2003; Maris et al., 2007). NBM can result from *MYCN* oncogene amplification, which is associated with poorest prognosis, 11q deletions, or *TERT* rearrangements followed by additional mutations in genes involved in RAS-MAPK signaling, in neuritogenesis, or in chromatin remodeling (Johnsen et al., 2019).

A recent genome-wide association study identified a NBM risk locus at 6p22.3, characterized by a cluster of single-nucleotide polymorphisms within intron regions of the long non-coding RNA (lncRNA) gene *CASC15* (also known as *LINC00340*), whose low levels have been previously associated to high-risk NBM (Russell et al., 2015). Interestingly, CASC15-depleted NBM SH-SY5Y cells display increased proliferation, migration, and invasion due to the persistent activation of the CHD7/SOX9 pathway (Mondal et al., 2018). CHD7/SOX9 signaling is necessary for NC cell specification and migration (Bajpai et al., 2010), and trunk NC cells derived from CASC15-depleted hESC exhibited a failure in neuronal differentiation due to increased expression levels of both SOX9 and CHD7 (Mondal et al., 2018). Consistent with this, the persistent activation of CHD7/SOX9 signaling in *CASC15*-depleted NBM cells impairs retinoic acid-induced differentiation in neurons, and this is reflected in their transcriptomic profile, with increased levels of NC-related genes and decreased levels of neuronal differentiation genes. Among these, several NC-related semaphorins are upregulated (*SEMA3C*, *3D*, and *6A*), whereas the neuronal-associated semaphorin *SEMA5B* is downregulated (Figure 3; Mondal et al., 2018). Interestingly, SEMA3C has been previously demonstrated to be fundamental for NC development (Schulz et al., 2014b), and a recent tumor transcriptional profiling showed that NBM invasiveness is induced by the shutdown of SEMA3C, which functions as a pro-cohesion autocrine signal to constrain the tumoral mass (Delloye-Bourgeois et al., 2017).

### Glioblastoma

Glioblastoma is one of the most lethal forms of adult brain cancer and is the most common malignant high-grade glioma. GBM tumors are mainly located in the cerebral hemisphere (80%) and in the cerebellum (< 5%) and are characterized by microvascular hyperplasia, cell proliferation, necrotic foci, and the appearance of peculiar pseudopalisading cells (Zhang et al., 2020).

Glioblastoma can be divided in four distinct molecular subtypes: proneural, neural, classical, and mesenchymal (Louis et al., 2016). According to the TCGA, proneural GBM exhibits higher levels of CHD7 compared to other GBM subtypes, although CHD7 expression does not significantly correlate with patient prognosis (Boyd et al., 2019; Machado et al., 2019).

A recent work showed that CRISPR-Cas9 mediated knockout of *CHD7* in LN-229 GBM cells leads to decreased growth rate and invasion both *in vitro* and in orthotopic xenografts, ameliorating mice survival. Inversely, CHD7 overexpression in low-CHD7-expressing GBM cells (LN-428) increased cell motility, growth, and invasiveness (Machado et al., 2019). Among downstream targets that have been perturbed by either CHD7 deletion or overexpression, class 3 semaphorins significantly emerged. SEMA3F and SEMA3G are upregulated with increased CHD7 expression, while SEMA3D and SEMA6B levels are increased in the absence of CHD7 (Machado et al., 2019; Figure 3).

SEMA3G was also associated with decreased invasion and motility in U251MG GBM cells (Zhou et al., 2012). These seemingly contrasting data for SEMA3G may be explained by the high molecular heterogeneity of GBM cell lines and by the fact that high-CHD7-expressing cells specifically correspond to cells not expressing CD133, a marker of brain tumor-initiating cells. In addition, the expression of SEMA3A, 3D, and 3F inhibited angiogenesis and the growth of subcutaneous tumors derived from U87MG GBM cells (Sabag et al., 2012). However, in a more recent study, SEMA3A was highly enriched in GBM compared to non-neoplastic brain tissues, and function-blocking antibodies against SEMA3A have been developed and successfully tested for inhibition of tumor growth (Lee et al., 2018), suggesting that SEMA3A can also act as a promoter of tumor progression depending on the tissue context. Thus, further studies will be needed to clarify the role of SEMA3A in GBM progression.

Finally, a recent report demonstrated that hypoxic microenvironments within the GBM tumor mass may repress CHD7 expression in glioma-initiating cells and, as a response, induce angiogenesis. In the same article, low CHD7 was associated with increasing glioma grade and poor patient prognosis (Boyd et al., 2019). In the same article, Boyd et al. (2019) profiled the transcriptome of CHD7-depleted cells and observed a decreased expression of SEMA3D, a previously identified anti-angiogenetic factor (Sabag et al., 2012), in contrast with increased levels of SEMA4A and SEMA3C, which is positively associated with glioma malignancy (Vaitkiene et al., 2015).

## Conclusion

In conclusion, multiple lines of direct and indirect evidence suggest the existence of genetic and molecular interactions between CHD7 and semaphorins during both development and tumorigenesis. In particular, this interaction appears to be important in the patterning of CNS structures and subtypes of neurons. The CHD7–semaphorins axis also appears fundamental in cardiac NC delamination and migration and, as a consequence, heart development. Now, emerging data are beginning to highlight the contribution of CHD7–semaphorins network in the pathogenesis of brain tumors. However, the regulatory mechanisms by which CHD7 controls semaphorins, as well as which and how downstream signaling cascades are modulated, are still to be elucidated. Studies aimed at filling such gaps in knowledge will be extremely meaningful to counteract, in the future, the adverse effect of deregulation of the CHD7–semaphorins network.

## Author Contributions

AL and RO critically collected literature material, planned the review flow, and wrote the main text. AP prepared the figures and figure legends. VM and CG contributed to editing and revision. AC and AF critically contributed to the writing, editing, and revision of the whole review. All authors contributed to the article and approved the submitted version.

## Conflict of Interest

The authors declare that the research was conducted in the absence of any commercial or financial relationships that could be construed as a potential conflict of interest.

## Footnotes

1 https://portal.gdc.cancer.gov/
